# Cotard and Capgras Syndrome in a Patient with Treatment-Resistant Schizophrenia

**DOI:** 10.1155/2021/6652336

**Published:** 2021-01-23

**Authors:** Joshep Revilla, Stephanie Aliaga, Antonio Lozano-Vargas

**Affiliations:** Psychiatry Section, Department of Medical Sciences, Universidad Peruana Cayetano Heredia, Lima, Peru

## Abstract

The presentation of both Cotard and Capgras syndromes is uncommon in schizophrenia. We present a case of a 23-year-old male with the diagnosis of schizophrenia with Cotard syndrome who later developed Capgras syndrome. By persisting significant symptoms despite the use of two antipsychotics, he was given the diagnosis of treatment-resistant schizophrenia, and his symptoms improved with clozapine. This is one of the few cases of Cotard and Capgras syndromes in a patient with schizophrenia.

## 1. Introduction

The presentation of both Cotard and Capgras syndromes is uncommon in schizophrenia, and a few cases have been reported. Cotard syndrome was first described by Jules Cotard in 1880, and one of the characteristics is the nihilistic delusions that are related to denying the existence of oneself or the world. Capgras syndrome was described in 1923 by Capgras and Reboul-Lachaux and is characterized by the delusion that the individual or family members have been replaced by substitutes [1]. We report the case of a patient with treatment-resistant schizophrenia who had symptoms of both syndromes.

## 2. Case Presentation

**Figure 1:**
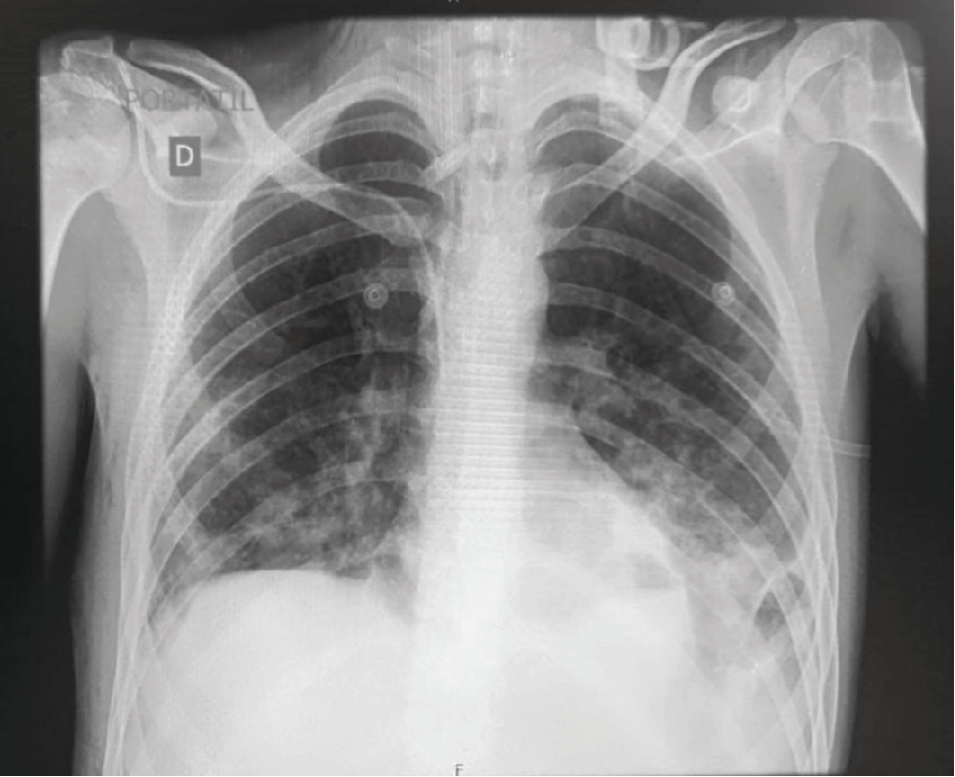
Frontal chest radiograph showing consolidation of both lungs with increasing density towards the lung bases, predominantly the left lung base.

**Figure 2:**
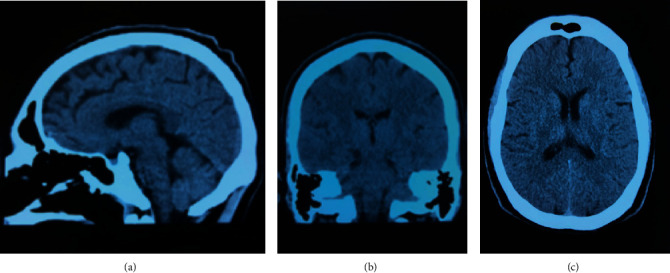
Noncontrast computed tomography of the head.

R. is a 23-year-old, male, with a family psychiatric history of psychotic disorder and whose symptoms started at 13 with social isolation and poor hygiene. At 15, he became aggressive and suspicious, and risperidone 3 mg was prescribed daily; then, auditory hallucinations developed, so the antipsychotic was increased to 4 mg daily. However, he subsequently presented on numerous occasions to the emergency room (ER) with aggressive behavior and persecutory delusions. When he was 18, disorganized behavior and delusions of prejudice were added; for this reason, sulpiride 400 mg and olanzapine 10 mg daily were prescribed but without good response. Then, he presented with nihilistic delusions concerning his existence “I am dead, I died in 2012,” nihilistic delusions related to his body “I do not have a heart,” hypochondriacal delusions “my organs are not working,” delusions of guilt “I am taking the blame,” and suicidal ideation. For these reasons, he was again taken for his dad to the ER. In the mental examination, he was agitated, asking people to kill him. The PANSS upon admission of the patient was 125. During the hospitalization, he continued with the suicidal ideation, and the initiation of electroconvulsive therapy was recommended. The patient received 25 sessions of electroconvulsive therapy with partial improvement in the psychotic symptoms. His previous medication was changed to quetiapine 900 mg daily, without improvement. He was given a diagnosis of treatment-resistant schizophrenia, and clozapine was started. During hospitalization, the patient started refusing food, and a nasogastric tube was inserted. On one occasion, he became unwell, developing aspiration pneumonia (Figure 1) and spent 15 days in intensive care. As his medical condition subsequently improved, he said “I don't have parents, the people who raised me are impostors, they look like my parents, they will be angels, my parents buried me and I never knew about them.” In the neurological physical exam, the patient was alert, oriented to person and time. He mentioned he was in purgatory. The speech was normal. The muscle strength was 5/5 bilaterally, with no motor deficits. The sensation was intact bilaterally. Reflexes are 2+ bilaterally. Brain computerized tomography scan showed no intra- or extra-axial fluid, hemorrhage, or mass; the ventricular size was normal, and the grey-white differentiation was preserved (Figure 2). The complete blood count, liver function test, creatinine, and glucose levels were within normal values. He later transferred back to the mental health hospital. The clozapine dose was gradually increased to 650 mg daily with an improvement of the auditory hallucinations, the suicidal ideation, and the symptoms of Cotard and Capgras syndrome. PANSS at the discharge was 51. A weekly normal absolute neutrophil count was obtained. The last WBC was 6500 cells/mcL and the ANC 4290 cells/mcL.

## 3. Discussion

In this case report, we present a patient with schizophrenia with Cotard syndrome who later developed Capgras syndrome. The association of Cotard and Capgras delusions in the same patient is extremely rare [2].

Cotard and Capgras delusions can also be understood as the one-stage or the two-stage model [3]. In the first one, the experiential, delusions are elucidated as a normal rationalization of the unusual perceptual experience. In the second one, the inferential, delusions are considered as an abnormal rationalization of the unusual perceptual experience. In this case, they may represent the way a person tries to attribute negative events to external causes (as in the case of Capgras that is accompanied by delusions of persecution) or internal causes (as in the case of Cotard in the setting of a depressive disorder) [4].

In our case, the Cotard syndrome did not improve with the electroconvulsive therapy (ECT), successful treatment in cases of melancholia, or psychotic depression [5]. The reasons for this could include that the Cotard syndrome appeared in the psychotic setting, the early onset of the disorder alongside his strong genetic load (both parents having features of schizophrenia). Our patient had persisting significant symptoms despite the use of two antipsychotics suggesting treatment-resistant schizophrenia, so the treatment was progressively changed to clozapine.

Through the observation of this case, we conclude that the recognition of symptoms of Cotard and Capgras syndrome in schizophrenia is important for the assessment of appropriate treatment.
